# Promoting workplace stair climbing: sometimes, not interfering is the best

**DOI:** 10.1186/s13690-016-0170-8

**Published:** 2017-01-09

**Authors:** Andreas Åvitsland, Ane Kristiansen Solbraa, Amund Riiser

**Affiliations:** Sogn og Fjordane University College, Faculty of Teacher Education and Sport, Box 133 – N 6851 Sogndal, Norway

**Keywords:** Physical activity, Public health, Active transport, Nudging, Quasi-experimental

## Abstract

**Background:**

Stair climbing is a vigorous activity and can lead to several health benefits. Studies seeking to increase stair climbing in various public locations have shown positive effects, while results from similar studies conducted in the workplace are inconclusive. This study examined stair climbing in the workplace, and monitored effects from a single- and a combined intervention. Interventions were inspired by nudging, the libertarian method of influencing behavior.

**Methods:**

By quasi-experimental design, stair- and elevator traffic in two office buildings was monitored preceding-, during- and following interventions with stair leading footprints alone, and combined with stair-riser banners. Chi square tests were applied to determine differences between baseline and the subsequent periods. Web-based questionnaires were distributed after follow-up period.

**Results:**

Elevators and stairs were used 45 237 times, of which 89.6% was stair use. Intervention site stair climbing at baseline (79.0%) was significantly reduced with footprints (-5.1%, *p <* 0.001), and footprints with stair-riser banners (-5.7%, *p <* 0.001) while baseline stair climbing at the control site (94.2%) remained stable (*p >* 0.027).

**Conclusions:**

Stair climbing was significantly reduced during the intervention periods. Use of stair leading footprints alone, or combined with stair-riser banners in an attempt to influence stair climbing may be ineffective, or cause a negative reaction, when applied in a workplace with a pre-existing high amount of stair climbing.

## Background

Physical inactivity is a major risk factor for non-communicable diseases (NCDs) [1]. Being physically active is associated with reduced risk of cardiovascular disease [2], type 2 diabetes [3], colon cancer [4] and obesity [5]. The recommended 150 min per week of moderate to vigorous physical activity [6] is only fulfilled by 32.0% of Norwegian adults [7] while inconclusive evidence suggests the equivalent portion in the USA to be somewhere between 8.2% [8] and 57.0% [9]. When North-American adults mention “lack of time” as a main reason for inactivity [10], while spending half their waking hours at work [11], the workplace should be considered an attractive arena for increasing physical activity levels. This could be done by increasing workplace stair climbing. Stair climbing expends between 8.6 METs [12] and 9.6 METs [13], and can therefore be categorized as a vigorous activity. Seven daily minutes of vigorous physical activity has been associated with a 62.0% decrease in coronary death [2], thus seven daily minutes of stair climbing should provide the same benefit. Stair climbing has been associated with higher peak VO_2_ [14]_,_ lower blood pressure [15]_,_ improved fitness [16], and is also timesaving, compared to elevator [17, 18]. There exist several studies specifically designed with the purpose to increase workplace stair use. Some have not been able to obtain the desired effect [19–22], while others have managed to show a significant increase [23–25]. Similar interventions have also been carried out in public locations, such as shopping centers [26], train/tram stations [27], airports [28] and universities [29], and according to Eves & Webb [30], interventions in these settings are often more successful than in the workplace. The typical intervention tactic is to place a sign at the point of choice between stairs and elevator, displaying a message or image. Russel, Ryan & Dzewaltowski [31] used a deterrent message, which gave a small, but significant increase in stair climbing. Eckhardt, Kerr & Taylor [32] compared general health related messages to specific health related messages and found specific messaging to be significantly more effective. Webb & Eves [33] placed specific health- and calorie related messages on stair risers and were able to increase stair climbing significantly. Placing messages on the stair risers instead of point-of-choice posters, was first demonstrated by Kerr, Eves & Carrol [34], who concluded that this new message format was superior to posters. Other tested interventions are music and art [25, 35] and reward based programs [36], all of which seem to increase stair use to some degree. In the efforts to change general behavior, positive feedback has been effective [37] and is known to have a more positive influence on motivation, as compared with external rewards [38]. This approach has also been used by Lewis & Eves [39] in a study aimed to increase stair use in a public access setting. No other studies using the approach of positive feedback to increase stair climbing could be found, presenting the possibility of exploring this gap in knowledge. Footprints leading towards the stairs are used in Norway by various health organizations to promote stair climbing. This intervention has only been tested once before [22], in a study which yielded negative results. Because of its current use in Norway, and lack of positive evidence, it is in need of re-testing. Previous research gives reasonable expectations towards a greater effect when combining interventions [24], and the combination of footprints and positive feedback has, to our knowledge, never been tested. All aforementioned interventions are created to influence decision making, while people retain their opportunity to choose freely. This is called nudging, and is defined by Thaler & Sunstein [40]. Successful ways of influencing employees, otherwise sedentary at work, to choose stairs over elevator means a major potential increase in physical activity levels. A natural consequence would be a decrease in NCD incidence in a cost-effective, available and timesaving way. Based on the mixed findings in this research field, the primary aim of the present study is to evaluate the effectiveness of stair-leading footprints in a solo intervention, and in combination with stair-riser banners providing positive feedback, to increase stair climbing in an office workplace setting. The secondary aim is to assess opinions of the interventions and of stair use in general.

## Methods

### Design

**Fig. 1:**
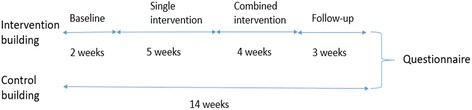
Stages of intervention and duration

The present study used a quasi-experimental time-series design. Stair and elevator use were monitored simultaneously in two office buildings, from early September to mid-December. Which building would receive the intervention and which would function as a control, was decided by coin toss. Both buildings had four floors. In the intervention building, stair and elevator proximity to the main entrance, measured four and six meters, respectively. The equivalent distances in the control building were fourteen and thirteen meters. In the intervention building, stairs and elevator were located next to each other, while in the control building; stairs and elevator were ten meters apart, separated by a wide foyer. Two weeks of baseline monitoring preceded a five-week intervention with stair leading footprints. This intervention was reinforced by adding stair-riser banners, and the combined intervention was displayed during four weeks, after which, the footprints and banners were removed. Subsequently, follow-up monitoring lasted three weeks. See Fig. 1 for a graphic overview. After the fourteen-week monitoring period, a web-based questionnaire was distributed via e-mail to all employees in both buildings.

### Sample

The office buildings are located in a small town in western Norway, with a population <2500. The intervention building accommodated the regional social services and contained approximately 140 employees. The control building accommodated the County Governor and contained approximately 170 employees. All registered counts from the monitoring period were included in the results. Human resource management in both buildings approved of the monitoring, as no health variables are collected, and no person can be recognized in the results. They also informed that mostly employees frequented the stairs and elevators, but they could receive occasional visitors.

### Measures

Infrared bi-directional people counters (Immotion, Sensor Development International, Dalen, Netherlands) were placed in the ground floor stair flight and elevator entrance of both buildings, 125 cm ± 1 cm from the floor. Structural differences in stairwells and elevators caused the distances between receiver and transmitter at each site to vary with 152 cm at the farthest, to 95 cm at the shortest. The outcome variables are ascent from-, and descent to the ground floor, by either stairs or elevator. The counters monitored at all time, providing counts also outside normal work hours, throughout the fourteen weeks. After the follow-up period, a web-based questionnaire (Questback, New York, USA) was distributed to all employees via e-mail. The questionnaire consisted of open-ended and closed questions. Respondents were questioned about their stair habits and if the project had affected them in any way. They were also asked how many floors they would climb by stairs, before choosing elevator.

### Intervention

**Fig. 2:**
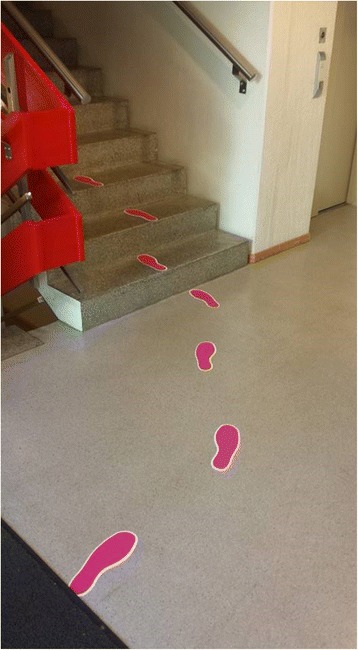
The stair leading footprints, as seen from the main entrance

**Fig. 3:**
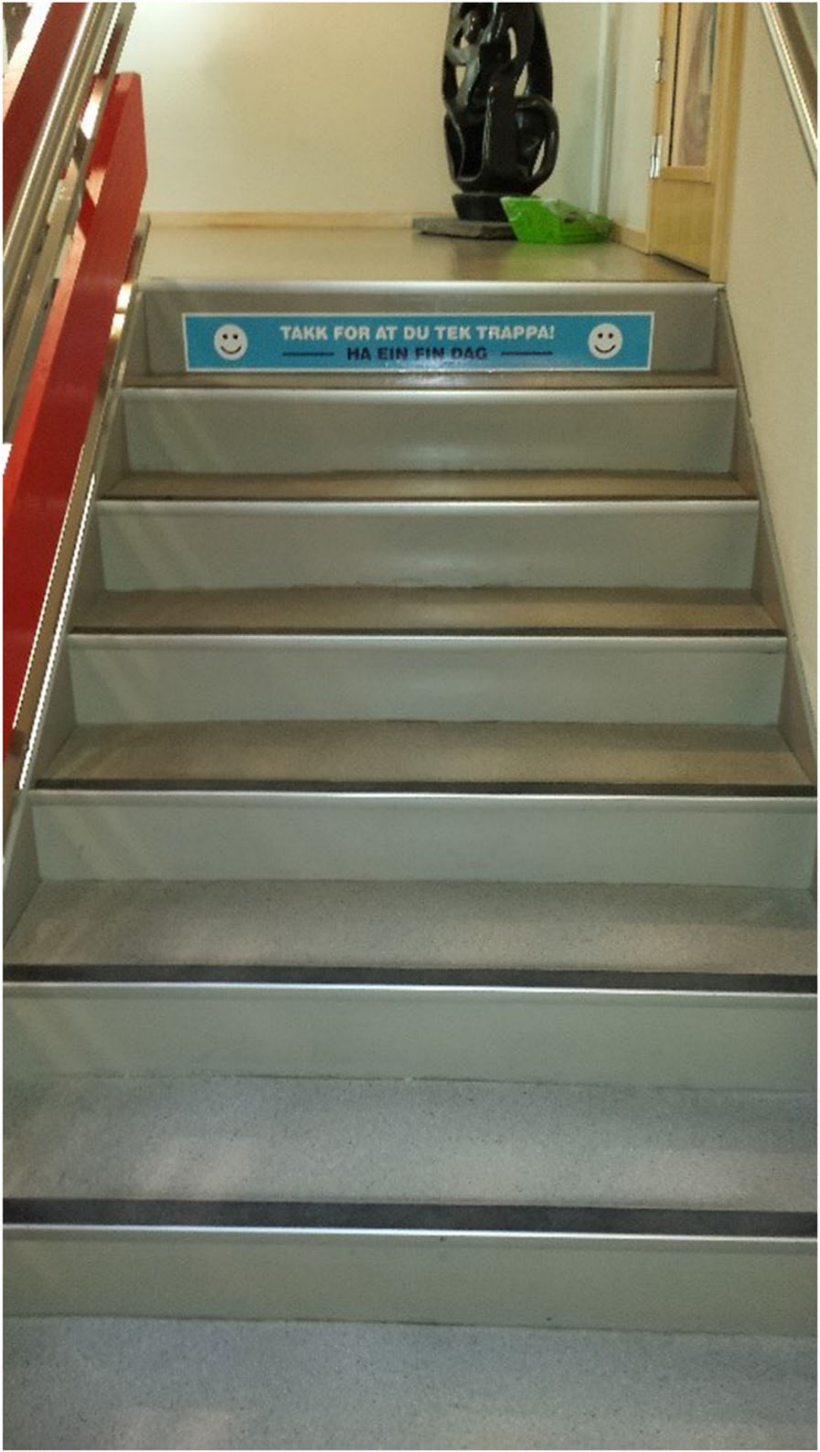
The stair-riser banners, as seen from mid-stairwell, leading to the fourth floor

The first intervention consisted of pink footprints with white edges, leading from inside the main entrance to the closest stairs (Fig. 2). Length of the footprints measured 27 cm and distance between the heels of each footprint was 66 cm. The combined intervention was employed by adding stair-riser banners containing a positive feedback message, placed at every top stair riser before reaching the next floor (Fig. 3). The banners displayed a light blue background, smiley faces on each side and the text (translated from Norwegian), “Thanks for taking the stairs. Have a nice day”. All intervention materials were removed before follow-up monitoring. The project was performed within the guidelines of the Helsinki declaration.

### Analysis

Reliability testing of the monitors was conducted by the means of a trial person passing all counters fifty times in both directions, in their place of set up. Analyses of the results were performed in IBM SPSS Statistics 23 (IBM, Armonk, New York, USA), where chi square tests were used to examine the difference in stair climbing and elevator ascent, by comparing baseline counts with counts from each subsequent period. Data are reported as complete counts from each period. The same testing procedure was used for examining the difference between stair climbing and stair descent, except the results were reported as complete counts from the entire project duration. The Bonferroni adjustment of multiple testing [41] set the significance level at <0.017. Questions 1, 3 and 5 in the questionnaire were open ended, but the answers we received led us to code them for specific themes (Table 2) with the purpose of sorting them into related option categories. These categories were based on the wording and themes of the answers, and were formed after all answers were collected. Chi square tests were applied to compare the control- and intervention group’s answers in the questionnaire.

## Results

### Effects of intervention

From the reliability testing of the monitors, we calculated the accuracy to be between 93.0% and 99.0%. Throughout fourteen weeks of monitoring, the intervention site counters made 17 400 registered ascents from-, and descents to the ground floor, by either stairs or elevator. The corresponding amount registered at the control site was 27 831. The complete results in stair use can be viewed in Table 1.

**Table 1:** Stair use at both sites throughout fourteen weeks

	Intervention building		Control building	
	Stair climbing	Stair descent	Stair climbing	Stair descent
Baseline (2 weeks)	79.0% (*n =* 940)	91.3% (*n =* 1105)	94.2% (*n =* 1920)	94.9% (*n =* 2052)
Footprints only (5 weeks)	73.9% (*n =* 2353)†	89.0% (*n =* 2761)†	94.6% (*n =* 4394)	94.9% (*n =* 4566)
Footprints and SRBs* (4 weeks)	73.3% (*n =* 1825)†	90.2% (*n =* 2194)	92.7% (*n =* 3617)	92.9% (*n =* 3873)†
Follow-up (3 weeks)	75.0% (*n =* 1421)	90.8% (*n =* 1713)	95.4% (*n =* 2813)	94.9% (*n =* 3001)

* SRBs = Stair-riser banners

† Significant decrease from baseline (*p <* 0.017)

**Fig. 4:**
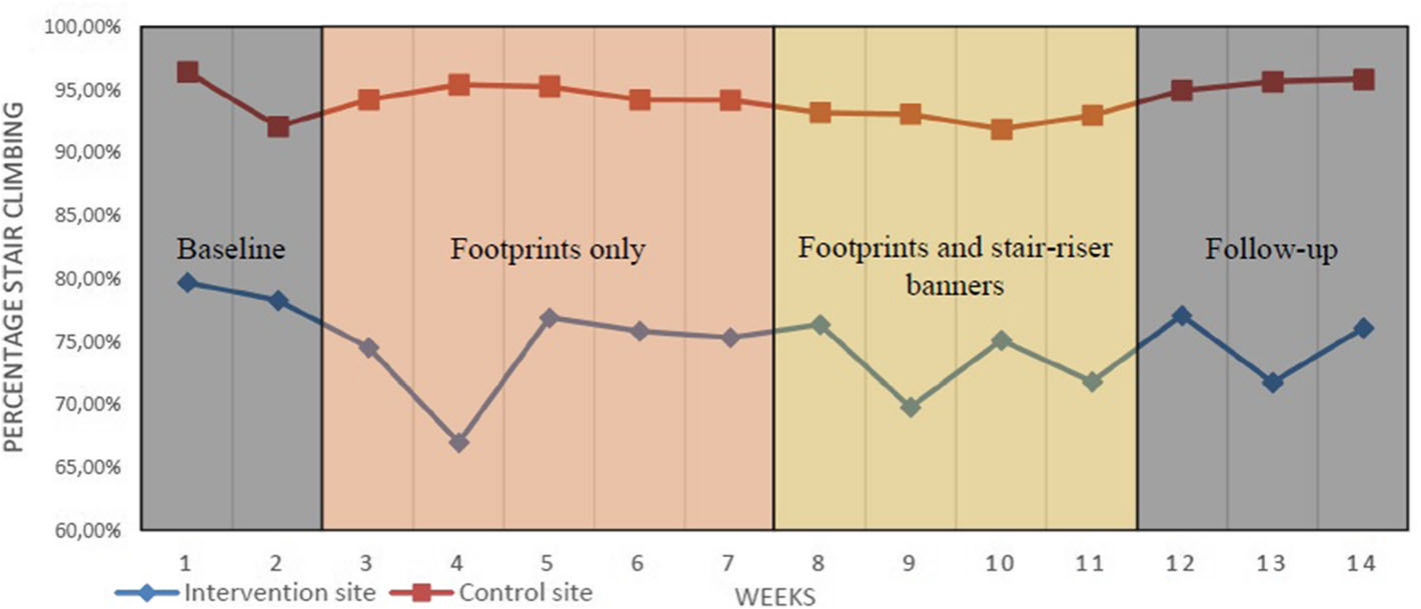
Stair climbing development in intervention site and control site

Stair climbing at the intervention site decreased from 79.0% at baseline to 73.9% (*p <* 0.001) in the first intervention period, and 73.3% (*p <* 0.001) in the combined intervention period. Intervention site stair climbing at follow-up was 75.0% (*p =* 0.019), a non-significant decrease, when using the Bonferroni adjustment. The week-by-week progression in stair climbing at both sites is displayed in Fig. 4. Overall stair climbing in the intervention building averaged 15.0% lower (*p <* 0.001) than stair descent, while there was no difference in the control building (*p =* 0.653). The only change in the control building was a significant decrease of 2.0% (*p =* 0.002) in stair descent, during the combined intervention period. There was no significant change during the remaining periods.

### Questionnaire

Response rates from intervention- and control building were 27.9% (*n =* 39) and 45.9% (*n =* 78), respectively. The qualitative answers from questions 1, 3 and 5 were coded for themes, and placed in appropriate option categories (Table 2). The remaining answers were quantitative and could easily be analyzed. When asked about their main reason for choosing stairs, exercise related answers were the most frequent from the control- (50.0%, *n =* 44) and the intervention participants (45.0%, *n =* 18). When intervention participants were asked about the counters, one person “wanted to take the elevator in spite”, while another said the project “felt like surveillance”. A few respondents were irritated by the stair-riser banners and when asked about the intervention, one respondent “did not like nagging” while another called the banners “provoking”. One respondent said “the banners gave base for some workplace discussion”, in which some thought they were a good idea and others felt that they were intrusive. Two respondents wondered “who out there is thanking me” and called it “disturbing” to be thanked by someone unknown for taking the stairs. Others were more positive, and 10.3% (*n =* 4) of participants mentioned that the interventions were “fun” or “funny”. One person answered that the intervention made it a more “positive experience to walk the stairs” and another person thought the greetings on the stair-riser banners were “a nice way to start the day”. Within intervention participants, 89.5% (*n =* 34) would climb four floors or more, before choosing the elevator, while in the control building, 97.4% (*n =* 75) would do the same. Complete results from the questionnaire are presented in Table 2.

**Table 2:** Complete questionnaire with results

Questions	Options/categorical themes	Int. % (n)	Con. % (n)	P
1.What is the main reason why you use/do not use the stairs at the workplace?	Mentions exercise related reasons	45% (18)	50% (44)	0.60
	Mentions health related reasons	12.5% (5)	6.8% (6)	0.28
	Mentions efficiency related reasons	7.5% (3)	15.9% (14)	0.19
	Mentions habit related reasons	25% (10)	20.5% (18)	0.56
	Other reasons	2.5% (1)	2.3% (2)	0.93
	No stairs: bad knees, don’t want to be out of breath	7.5% (3)	0% (0)	0.01
	Maybe elevator: because carrying heavy objects	0% (0)	4.5% (4)	0.17
2.Did you notice the counters in the stairwell and the elevator entrance?	Yes	79.5% (31)	35.5% (27)	<0.001
3.Mention a way the counters influenced you, or why they did not influence you.	Always take the stairs anyway	51.4% (19)	7.2% (5)	<0.001
	Increased awareness of stair use	5.4% (2)	1.4% (1)	0,24
	Not influenced, no mention if stair user	8.1% (3)	31.9% (22)	0.006
	Did not notice the counters	13.5% (5)	58% (40)	<0.001
	Always use elevator anyway	2.7% (1)	1.4% (1)	0.65
	Was reminded of stair use	8.1% (3)	0% (0)	0.01
	Negative reaction, spiteful, annoyed, skeptical	10.8% (4)	0% (0)	0.01
4.Did you notice the footprints and the stair riser banners?	Both	76.9% (30)		
	Only footprints	17.9% (7)		
	Only stair riser banners	2.6% (1)		
	No	2.6% (1)		
5.Mention a way the footprints and/or the stair riser banners influenced you, or mention why they did not influence you.	Always take the stairs anyway	28.2% (11)		
	Was influenced/felt pulled towards stairs	5.1% (2)		
	Increased awareness of stair use	2.6% (1)		
	Made stair walking a positive experience	20.5% (8)		
	People felt “led” to the stairs by the footprints	10.3% (4)		
	Thought they were fun/funny	10.3% (4)		
	Created a basis for discussion in the workplace	7.7% (3)		
	Not influenced, no mention if stair user	7.7% (3)		
	Negative reaction, spiteful, annoyed, dislike	7.7% (3)		
6.Has your stair use outside the workplace…	Increased	5.1% (2)		
	Decreased	0% (0)		
	Remained the same	87.2% (34)		
	Do not know	7.7% (3)		
7.How many floors are you willing to climb by stairs before choosing the elevator?	1	2.6% (1)	0% (0)	0.15
	2	0% (0)	1.3% (1)	0.48
	3	7.9% (3)	1.3% (1)	0.07
	4	10.5% (4)	23.4% (18)	0.10
	5	26.3% (10)	23.4% (18)	0.72
	6	13.2% (5)	19.5% (15)	0.40
	7	7.9% (3)	3.9% (3)	0.98
	8	5.3% (2)	7.8% (6)	0.61
	More than 8	26.3% (10)	19.5% (15)	0.40

*Int* Intervention, *Con* Control, *N* number of answers, *%* percentage of all answers

## Discussion

The present study has demonstrated a negative effect in stair climbing from stair-leading footprints alone and in combination with stair-riser banners displaying positive feedback. The results are comparable with the findings of Coleman & Gonzalez [21], who reported negative effects with male participants in both a library and an office setting. They suggested the reason to be a ceiling effect, in which case the baseline values would be too high for any increase to occur. However, a ceiling effect in the present study is somewhat unlikely, since stair descent in the intervention building averaged 15.0% higher than stair climbing throughout the monitoring period. In addition, average stair climbing in the control building was 19.0% higher than in the intervention building and this site displayed no difference between stair climbing and descent. In a similar study by Eves, Webb, Griffin & Chambers [24], results showed significantly higher increase in stair climbing, when using two simultaneous interventions, rather than a single intervention. The two interventions were placed at the point of choice and inside the stairwell, similar to the intervention placements in the present study. However, while they used verbal informational posters, the present interventions are non-verbal encouragements and non-informational positive reinforcements. It seems information may be superior to the present intervention approach, as informational posters have been effective in the workplace on multiple other occasions [23, 42]. The footprints were selected as intervention because they are used as an effect to promote physical activity by various health organizations in Norway. However, to our knowledge, the only published study that has tested footprints as an intervention in the past also found negative effects [22], despite baseline stair use being lower than 16.0%. This strengthens the results of the present study and it seems the Norwegian health organizations should consider discontinuing their use of footprints. To our knowledge, stair-riser banners with a positive feedback message have not been tested in the past. The present results suggest that how a message is presented may be less important than the message itself. Kerr, Eves & Carrol [34] judged stair-riser banners to be superior to point-of-choice posters to increase stair use, and they have proven effective a number of times [27, 33], but they may also be ineffective [43]. Nevertheless, the presented messages in past studies have almost always been calorie- or health related. The present stair-riser banners involved positive feedback, inspired by Schultz, Nolan, Cialdini, Goldstein, & Griskevicius [37] who were able to decrease participants’ use of electricity with smiley faces. Though it has been established that positive feedback may enhance motivation [38], the opposite happened in the present study. The reason for this difference may be that the present intervention message was the same for everyone who went up the stairs. In the former experiment, participants were given personal information of their electricity use compared with their neighbors, and a smiley face if their electricity use was below average. Perhaps personalized social comparisons would have increased stair use in the present study, but that would have involved some form of self-reporting or personalized counting system, in which the participants register each time they climb a flight of stairs, and receive some form of feedback at the end of the week. In any case, further testing of the stair-riser banners in sites with lower baseline stair use would be of interest, before dismissing them completely as an intervention. Throughout the fourteen weeks of monitoring, stair climbing at the control site averaged 19.0% higher than the intervention site. When comparing the design of the two buildings, these results are in accordance with previous research. Stairs and elevator are farther apart in the control building than in the intervention building, which can increase stair use [44]. The stairs in the control building were located openly in a large entrance foyer, leaving it exposed to natural lighting, which may also increase stair use [45]. In comparison, the intervention stairwell was without windows. The present study is the first of its kind to be performed in Norway, and may present the image of Norwegians as above average physically active people, because of the high portion of stair climbers. However, previous research has found the people of the present county to be more active than the country average [46], which could help explain the unprecedented amount of baseline stair climbing. According to a review by Eves & Webb [30], average baseline stair use in the workplace is only 20.9%, which is roughly ¼ of the baseline stair use in the present study. People of the present municipality is also the second highest educated in Norway [47], and it is well known that higher education is associated with physical activity levels above average [48]. Further research should be done on this topic, subjecting other populations to similar interventions. For some reason, the control building displayed a significant decrease in stair descent during the combined intervention period, but we suspect this to be a coincidence.

### Questionnaire

The questionnaire was distributed because it was decided that qualitative information was necessary for an extended understanding of the objective results. Answers from the questionnaire suggest that the decrease in stair climbing can be attributed to a few respondents who were irritated that someone would come to their workplace and try to influence their behavior. Previous research has suggested that the intervention message needs to be believable, to create motivation for increased stair climbing [24]. In the present study, the stair-riser banners are positive reinforcements from an unknown source, which may have caused them to be interpreted as insincere, and may be a reason for the negative responses. The questionnaire suggests that the participants in this study were quite conscious about the fact that they use stairs for exercise reasons. This is yet another supporting argument that the present population is more physically active than average, as well as being conscious about this behavior. Another finding that makes this population out of the ordinary is how many flights of stairs they are willing to climb. Previous research has shown that people are on average willing to climb less than four floors [20]. The present questionnaire reveals that when the option “more than 8” is calculated as nine, employees from both sites combined, are willing to climb an average of six floors, before choosing the elevator. On the other hand, in the present municipality, or county for that matter, buildings higher than four floors are not quite common. Anyone could say they would climb eight flights of stairs, but we do not know if this would be the case, were they given the opportunity. More studies are needed to establish if this is a tendency in the whole country, or if it only exists in the present municipality.

### Strengths and limitations

A limiting factor is the high percentage of stair users. This makes any increase difficult, and it is possible that effects would be different in a site with lower stair use. The strength of a quasi-experimental design is the ability to compare results to a control population, which several previous studies have failed to do [19, 26, 27, 49]. In addition, the present population groups are, despite the baseline differences, quite comparable: Both groups are inhabitants of the same small town and have typical sedentary desk jobs, in buildings, which share the same amount of floors. However, the results are less representative compared to results from a randomized controlled study. In further research on this topic, several buildings should be used and randomized, in order to diversify the results and investigate different work environments. Another improvement to the design would be to have one control group, one single intervention group and one combined intervention group. The reason would be to eliminate the possibility of the combined intervention results being influenced by the single intervention. Objective people counters have the advantage of being able to monitor at all time, which provides large amounts of count data, compared to monitoring by human observers. The disadvantage is inability to account for other variables, such as gender, age and weight, in order to adjust for said variables, or do sub-group analyses. Answers from the questionnaire suggest that the counters had been intrusive to the extent that people would take the elevator in spite, making it clear that some other form of hidden monitoring is preferred. However, only 35.5% (*n =* 27) in the control building noticed the counters, which suggests that their intrusiveness may have been exaggerated by intervention participants. The intervention building’s low response rate to the questionnaire is another weakness, prompting assumptions of representativeness to be treated with caution. The questionnaire still provides important knowledge of how the intervention was received, and is an appropriate supplement to the objective results.

## Conclusion

Both intervention periods resulted in significant decreases in stair climbing, a decrease that was not present during follow-up, when applying the Bonferroni adjustment. The results suggest that non-verbal and non-informative tactics in influencing stair climbing, may be ineffective, or cause a negative reaction, when applied in a workplace with a pre-existing high amount of stair climbing. Answers from the questionnaire suggest that the decrease is due to irritation among some employees, who did not like to be subjected to influence. In this case, the influence was telling people to do something they were already doing, which seems to have been interpreted as nagging, and resulted in spiteful behavior. Health promoters attempting to increase physical activity through stair use, should be more aware of what population they are trying to influence. Informational posters should be preferred as interventions, until further research unveils more effective methods. It may also be a question of building design, which architects, city planners and office managers should keep in mind.
